# DIGNITY THERAPY FOR OLDER ADULTS WITH CANCER: COMMUNION FACILITATES DIGNITY

**DOI:** 10.1093/geroni/igad104.2502

**Published:** 2023-12-21

**Authors:** Susan Bluck, Mary Kate Koch, Sophia Maggiore, Diana Wilkie, Harvey Chocninov, Carma Bylund

**Affiliations:** University of Florida, Gainesville, Florida, United States; University of Florida, Gainesville, Florida, United States; University of Florida, Gainesville, Florida, United States; University of Florida, Gainesville, Florida, United States; University of Manitoba, Winnipeg, Manitoba, Canada; University of Florida, Gainesville, Florida, United States

## Abstract

Nearly 600,000 older Americans die a cancer-related death annually. Maintaining dignity is central to their quality of life. Dignity Therapy (DT; Chochinov, 2005) was designed to preserve dignity despite health declines. Patients report benefits of DT but mechanisms have not been empirically investigated. Grounded in gerontological life review research, DT involves therapists guiding patients to tell their unique life story and leave a legacy. This study investigates level of communion in the life story told in DT as a mechanism for DT efficacy. Communion reflects personal strivings for warm and close relationships (McAdams, 1986). Participants were 204 older cancer patients receiving outpatient palliative care (Mage= 66 years, SD = 7.43, 65% female) who participated in DT. Session narratives were audio-recorded and reliably (κ=.71–1) content-analyzed for communion themes (McAdams, 2001). Participants completed pre-post measures, including the Dignity Impact Scale. Mediation analyses showed significance for the direct effect of Dignity Impact from pre- to post-test, B = .50(.06), p < .001 as well as for paths from pre-test Dignity to Communion, B = .20(.08), p <.01 and from Communion to post-test Dignity, B = .20(.06), p = .001. Sobel test for the mediation effect of Communion between pre- to post-test Dignity was z = 2.06(.02), p = .039; point effect = .04. Participating in DT is shown to increase older cancer patients’ feelings of dignity. That is partially explained by the extent to which these older adults tell a life story rich in communion, reflecting on loving, caring relationships, during DT.

